# Gut microbiome dysbiosis in hepatocellular carcinoma patients with persistent HCV viremia versus viral clearance: a cross-sectional study

**DOI:** 10.1186/s13099-025-00761-w

**Published:** 2025-11-14

**Authors:** Hany R. Hashem, Tasnem Yehia, Marwa Azab, Ali Abdellah, Ibrahim A. Amin, Mohammed Salah, Mohammed Ramadan

**Affiliations:** 1 Department of Microbiology and Immunology, Faculty of Pharmacy, Fayoum University, Al-Fayoum, Egypt; 2 Department of Microbiology and Immunology, Faculty of Pharmacy, Suez Canal University, Ismailia, 41522 Egypt; 3 Department of Microbiology and Immunology, Faculty of Pharmacy, Al-Azhar University, Assiut, 71524 Egypt; 4 Department of Microbiology and Immunology, Faculty of Pharmacy, Port-Said University, Port-Said, Egypt

**Keywords:** Gut microbiome, Hepatocellular carcinoma, HCV, Dysbiosis, Firmicutes/Bacteroidetes ratio, Microbial biomarkers, Direct-acting antiviral

## Abstract

**Background:**

Hepatocellular carcinoma (HCC) remains a lethal complication of chronic hepatitis C virus (HCV) infection, even after successful direct-acting antiviral (DAA) therapy. The gut microbiome influences hepatocarcinogenesis through the gut‒liver axis; however, the microbial signatures associated with HCC in DAA-treated patients are poorly defined. This study aimed to elucidate the patterns of microbiomes in HCV-treated patients who developed HCC, with a focus on bacterial diversity, differentially represented taxa, and their associations with clinical markers (FIB-4) and metabolic profiles as potential biomarkers.

**Results:**

A total of 138 participants were enrolled: 46 HCC patients with persistent HCV viremia (RHCC), 46 HCC patients with HCV eradication (THCC), and 46 healthy controls. RHCC patients exhibited pronounced dysbiosis, characterized by reduced alpha diversity (Kruskal–Wallis; H = 14.37, *p* = 0.00076) and an elevated Firmicutes/Bacteroidetes (F/B) ratio (1.55 vs. 1.05 in controls; Mann–Whitney U test, U = 87.32, *padj* = 0.00079). At the genus level, *Asteroleplasma* was significantly enriched in RHCC (log₂FC = + 2.8, *padj* = 0.008), whereas the butyrate-producing genus *Faecalibacterium* was depleted (log₂FC = − 2.1, *padj* = 0.006). Machine learning identified *Asteroleplasma, Moryella, Lachnoclostridium, Fournierella, Eubacterium xylanophilum, Succinivibrio*, and *Faecalibacterium* as the top classifiers of RHCC (AUC = 0.81). Functional profiling revealed a 58% reduction in butyrate synthesis (*padj* = 0.0032) and increased lipopolysaccharide biosynthesis (log₂FC = + 3.2, *padj* = 0.002) in RHCC, both of which correlated with clinical deterioration (FIB-4 scores, *r* = 0.62).

**Conclusions:**

Distinct gut microbial signatures distinguish HCC patients with persistent HCV viremia from those who achieve viral clearance, with implications for risk stratification and therapeutic targeting. The F/B ratio, abundance of *Asteroleplasma*, and functional pathway disruption (butyrate depletion) could serve as potential biomarkers for HCC progression. These findings underscore the influential role of the gut microbiome in hepatocarcinogenesis and its potential utility in personalized HCC management.

**Supplementary Information:**

The online version contains supplementary material available at 10.1186/s13099-025-00761-w.

## Background

HCC represents one of the most prevalent and lethal malignancies globally, accounting for nearly 90% of primary liver cancers [1, 2]. Its pathogenesis is intricately linked to chronic liver inflammation and fibrosis, with hepatitis C virus (HCV) infection recognized as a leading etiological factor. Despite improvements in surveillance and treatment, HCC has a poor prognosis, with 5-year survival rates under 20% in advanced stages [3, 4]. The development of HCC in HCV-infected individuals involves complex interactions between viral persistence, host immune responses, and environmental factors, creating an urgent need for improved risk stratification strategies [5].

HCV infection affects approximately 58 million people worldwide and continues to be a leading cause of cirrhosis and HCC [6]. Although DAAs achieve a sustained virologic response (SVR) in more than 95% of patients, evidence suggests that the risk of HCC persists, particularly in those with advanced liver fibrosis [7, 8]. This residual risk highlights the importance of identifying novel biomarkers that can be associated with HCC development following SVR. Recent studies have revealed that HCV infection alters host metabolic pathways and immune responses in ways that may persist after viral clearance, potentially contributing to hepatocarcinogenesis [9].

The gut microbiome is recognized as a critical modulator of liver health and disease through the gut-liver axis [10]. This bidirectional communication system involves microbial metabolites, pathogen-associated molecular patterns, and immune cell trafficking between the intestinal lumen and hepatic tissue. In healthy states, commensal bacterial inhabitants play integrative roles in maintaining intestinal barrier integrity and producing short-chain fatty acids (SCFAs), which exert anti-inflammatory and antifibrotic effects [11]. However, dysbiosis, characterized by reduced microbial diversity and altered composition, has been implicated in various liver disorders, including nonalcoholic alcoholic liver disease, fatty liver disease and HCC [12, 13].

The relationship between shifts in the gut microbiome and HCV-associated liver diseases has garnered increasing scientific interest in recent years [14]. HCV infection is strongly associated with gut barrier disruption, leading to increased intestinal permeability and microbial translocation [15]. These shifts may promote hepatic inflammation and fibrosis by activating pattern recognition receptors and the subsequent production of proinflammatory cytokines [16]. Emerging evidence also suggests that the gut microbiome may influence HCV treatment outcomes and post-SVR complications, including HCC development [17, 18]. However, the specific microbial signatures associated with HCC risk in HCV-treated populations remain poorly characterized.

The current understanding of post-DAA HCC risk factors relies primarily on clinical parameters such as liver stiffness, platelet count, and FIB-4 score [19]. While these markers provide some prognostic value, they lack the sensitivity and specificity needed for precise risk stratification. Recent studies have explored microbial biomarkers for liver disease progression, but few have focused specifically on the post-DAA population [20, 21]. This highlights a critical knowledge gap, as the gut microbiome may contain unique signatures indicative of ongoing oncogenic processes even after HCV clearance. Moreover, the differential microbial patterns between patients who develop HCC with and without persistent HCV viremia remain unexplored [22].

Notably, this study was conducted in the context of the Egyptian national campaign for HCV eradication, which has successfully treated millions of patients, offering a unique opportunity to investigate posttreatment microbial signatures in a population undergoing large-scale viral clearance [23]. Our study addresses these gaps through comprehensive profiling of the gut microbiome in HCC patients following DAA therapy, with a particular focus on comparing those with persistent HCV viremia to those who achieved SVR. This study integrates 16S rRNA sequencing with advanced bioinformatics and machine learning to uncover associations between bacterial taxa, metabolic pathways, and clinical outcomes, offering insights into how gut dysbiosis contributes to hepatocarcinogenesis after DAA therapy.

## Methods

### Study design and participant recruitment

This cross-sectional cohort study aimed to investigate changes in the gut microbiome composition among HCV-infected patients receiving DAA therapy and their association with HCC.

The study participants were stratified into three distinct groups according to HCV RNA status and hepatocellular carcinoma diagnosis: 1) HCC patients with confirmed HCV eradication following DAA therapy before study enrollment (THCC); 2) HCC patients with persistent HCV viremia (RHCC); and 3) well-matched healthy controls without HCC or HCV infection. The DAA-treated cohort demonstrated a median follow-up duration of 2 years post-treatment, ensuring the assessment of long-term microbial patterns rather than transient treatment effects.

The clinical investigations followed established methodological frameworks for hepatology microbiome research [24]. Our sample size determination, derived from power analyses of previous HCV microbiome studies [25], indicated that 40–50 participants per group would provide 80% power (α = 0.05) to detect moderate effect sizes (Cohen's d ≥ 0.5) in alpha diversity metrics.

Participant recruitment took place at the outpatient clinics of the Internal Medicine Department et al. Mabarrah Health Insurance Hospital in Zagazig, Egypt, between December 2023 and December 2024. The inclusion criteria were as follows: adult HCC patients (18–75 years) with documented HCV RNA status before and after DAA therapy; complete clinical records including specific DAA regimens (400 mg sofosbuvir plus 60 mg daclatasvir, with or without ribavirin following Egyptian national guidelines [26, 27]); and a minimum 2-year post-treatment interval to ensure the stabilization of treatment-related microbial changes.

Exclusion criteria were rigorously implemented to minimize confounding factors: HIV/HBV coinfection; recent antibiotic/probiotic use (within 90 days); active alcohol abuse (> 30 g/day); liver transplantation history; advanced cirrhosis (Child–Pugh C); uncontrolled diabetes; active dietary interventions; and immunomodulatory medication use. These criteria ensured that the observed microbial differences could be more confidently attributed to HCV and HCC status rather than extraneous factors.

## Ethics statements

The study protocol was approved by the Ethics Committee of the Faculty of Pharmacy, Suez Canal University (2023/0NH1), in accordance with the ethical principles of the Declaration of Helsinki [28]. All participants provided written informed consent.

### Hepatocellular carcinoma diagnosis and clinical assessments

All participants underwent comprehensive clinical assessments upon recruitment. HCC diagnosis followed international standards and Egyptian clinical guidelines, reflecting the high national burden of HCV-related HCC [29]. Clinical evaluations included liver imaging, laboratory tests, hematologic profiling, and noninvasive fibrosis assessment. Because formal fibrosis staging by biopsy or elastography was not consistently available at the time of HCC diagnosis, we utilized the FIB‑4 index and routine liver function tests (platelet count, ALT/AST, albumin, bilirubin, and the international normalized ratio (INR)) as alternative indicators of hepatic fibrosis and function. Advanced fibrosis was operationally defined as FIB‑4 > 3.25, a threshold with high specificity for advanced fibrosis in HCV cohorts. Abdominal ultrasound and transient elastography (FibroScan®, LSM > 12.5 kPa) were performed by certified operators (Echosens, France). Laboratory tests were conducted using the Roche Cobas 8000 system (Roche Diagnostics, Switzerland), and hematologic profiling was performed with Sysmex XN-series analyzers (Sysmex Corporation, Japan). Noninvasive fibrosis staging included FIB-4 scoring. Imaging-based diagnosis uses quadruple-phase CT or dynamic contrast-enhanced MRI, with lesion characterization based on the LI-RADS v2018 criteria [30, 31]. Nodules > 1 cm with arterial hyperenhancement and washout were considered diagnostic. In cirrhotic patients, AFP levels ≥ 200 ng/mL support the diagnosis [32]. Cirrhosis and portal hypertension were confirmed through consistent clinical and imaging findings.

### Stool sample processing and metagenomic sequencing

Fresh stool samples were collected in sterile containers, transported on ice packs, and stored at − 80 °C until processing. Microbial DNA was extracted in batches using the Qiagen DNeasy PowerSoil Kit (Cat. No. 12888–100) following the manufacturer’s protocol. The integrity of the DNA was verified using 1% agarose gel electrophoresis, and the concentration was defined as the samples' absorbance values at 260 and 280 nm using a Nanodrop ND-1000 spectrophotometer (ND-1000; Thermo Scientific, Waltham, MA, USA). The hypervariable regions (V3-V4) of the bacterial 16S rRNA genes were amplified using Forward Primer 5' TCGTCGGCAGCGTCAGATGTGTATAAGAGACAGCCTACGGGNGGCWGCAG and Reverse Primer 5 GTCTCGTGGGCTCGGAGATGTGTATAAGAGACAGGACTACHVGGGTATCTAATCC (The underlined bp are Illumina adapters) [33]. Sequencing of 16S rRNA amplicon libraries was performed on an Illumina MiSeq platform according to the manufacturer’s instructions (Illumina, San Diego, CA, USA) at IGA Technology Services (Udine, Italy). Negative extraction controls and PCR blanks were included in each batch and sequenced alongside samples to ensure that no contamination occurred during DNA extraction or amplification. This quality control step confirmed that the observed microbial profiles represented true biological signals rather than artifacts introduced during sample processing.

### Microbiome data analysis

Sequence processing utilized the DADA2 pipeline implemented in QIIME2 for denoising, chimera removal, and amplicon sequence variant (ASV) calling at 100% identity. Taxonomic assignment was conducted using the SILVA v138.1 reference database [34, 35] with a naive Bayes classifier trained at a 97% sequence identity threshold [36]. Alpha diversity metrics, Observed species (Obs), Chao1 (species richness), and the Shannon index (species richness and evenness), were calculated in QIIME2 after rarefying to 27,119 reads per sample (Additional file: Figure S1). Statistical comparisons of alpha diversity metrics and the F/B ratio across the three study groups were performed using the Kruskal–Wallis test. Pairwise comparisons between groups were conducted using the Mann–Whitney U test. All *p* values were corrected with Benjamini–Hochberg false discovery rate (FDR) for multiple testing to control the false discovery rate [37]. Beta diversity was assessed through Bray–Curtis distance (ASV-level composition) and both weighted and unweighted UniFrac distances (phylogenetic relationships), which were displayed through principal coordinate analysis (PCoA) plots. Statistical differences in microbial community structure between clinical groups were identified using permutational multivariate analysis of variance (PERMANOVA) with 999 permutations [38]. Differential abundance at the phylum and genus levels was analyzed using DESeq2 [39], which implements negative binomial Wald tests with Benjamini–Hochberg false discovery rate (FDR) correction. Microbial association analysis was performed using Maaslin2 (Multivariate Association with Linear Models), which applies a linear modeling framework to identify taxa significantly associated with clinical groups [40]. The genus-level OTU table was used as input data, and metadata included disease state-associated groups (Control, THCC, and RHCC), Age, and Sex. These variables were specified as fixed effects in the model to adjust for demographic confounding. Total Sum Scaling (TSS) normalization and log transformation were applied to stabilize variance and reduce compositional bias. Enterotyping was conducted using PAM clustering of Jensen-Shannon divergence distances from genus-level relative abundances, with the optimal cluster number determined by the silhouette width and Calinski-Harabasz index [41]. Microbial cooccurrences and associations with clinical parameters were assessed using Spearman correlation (*r* ≥ ± 0.3, *p* ≤ 0.05). The functional potential prediction utilized Tax4Fun with default parameters within the MicrobiomeAnalyst web platform [42–44]. The SILVA v138 database served as the reference for taxonomic and functional annotation at the 97% sequence identity threshold for nearest neighbor identification. The predicted functional profiles were mapped to Kyoto Encyclopedia of Genes and Genomes (KEGG) pathways, resulting in relative KO (KEGG Orthology) abundance levels that were used for subsequent functional diversity analyses [45]. The icons used in Figure S4 were obtained from Pixabay (https://pixabay.com) and is free for use without attribution under the Pixabay license (https://pixabay.com/service/license/).

### Statistical modeling and validation

The identification of potential microbial classifiers associated with HCC was achieved using machine learning methods validated in microbiome research [46]. A random forest model (randomForest package; 500 trees, max depth = 5) was constructed using genus-level relative abundance data as input features. All the statistical analyses were conducted in R (v4.4.2). The key genera distinguishing the study groups were identified using the Random Forest classifier within the MicrobiomeAnalyst web platform [42, 43]. To minimize overfitting, nested cross-validation was implemented (5 outer folds, 10 inner folds) for model training and hyperparameter tuning, and performance was evaluated through AUC, precision-recall, and calibration metrics (Additional file: Table S1; Figure S2). Additional validation included an independent 30% holdout set and 1,000 permutation tests to assess model robustness.

## Results

### Clinical and biochemical characteristics across study groups

The study cohort included three well-defined groups: HCC patients with persistent HCV viremia (RHCC, n = 46), HCC patients with HCV eradication (THCC, n = 46), and healthy controls (n = 46). Significant differences in liver function parameters were observed among the studied groups (Table 1). The control participants had normal liver function values. RHCC patients showed significant hepatic dysfunction, with significantly reduced albumin levels (3.0 ± 0.6 g/dL vs 4.3 ± 0.2 g/dL in controls; *p* < 0.001) and higher FIB-4 scores (6.55 ± 1.3 vs 1.13 ± 0.3; *p* < 0.001). THCC patients presented an intermediate phenotype, maintaining near-normal albumin (4.1 ± 0.4 g/dL) despite elevated AST (91 ± 29 IU/L).

**Table 1:** Demographic and biochemical characteristics of the recruited subjects

Parameter	Control (n = 46)	THCC (n = 46)	RHCC (n = 46)	p value
Age (years)	42.5 ± 12.4	45.5 ± 9.8	57 ± 6.2	0.072
Male sex	50%	62.5%	75%	0.421
Platelets (× 10^9^/L)	250 ± 25	218 ± 47	76 ± 24	< 0.001
AST (IU/L)	25 ± 5	91 ± 29	29 ± 7	< 0.001
Albumin (g/dL)	4.3 ± 0.2	4.1 ± 0.4	3.0 ± 0.6	< 0.001
Total Bilirubin (mg/dL)	0.8 ± 0.2	1.2 ± 0.3	1.55 ± 0.5	< 0.001
FIB-4	1.13 ± 0.3	6.04 ± 1.1	6.55 ± 1.3	< 0.001
AFP (ng/mL)	4.52 ± 1.68	272.05 ± 36.42	312.64 ± 56.1	< 0.001
INR	1.0 ± 0.1	1.08 ± 0.1	1.58 ± 0.3	< 0.001

*Data are presented as the means ± standard. Statistical analysis included the chi-square test for categorical variables and the Kruskal–Wallis test for continuous variables. *p* < 0.001 indicates statistically significant differences after multiple testing correction

### Comparative analysis of disease progression markers

RHCC patients exhibited the most advanced liver disease among the groups, with 100% meeting the criteria for cirrhosis, reflected in an elevated international normalized ratio (INR) of 1.58 ± 0.3 and significantly higher alpha-fetoprotein (AFP) levels of 312.64 ± 56.1 ng/mL; in contrast, THCC patients presented with residual hepatic damage, frequently presenting with advanced fibrosis, as indicated by a mean FIB-4 index of 6.04 ± 1.1. The liver function of the RHCC group was largely impaired and markedly more preserved than that of the RHCC group, as evidenced by a platelet count of 218 ± 47 × 10⁹/L, an international normalized ratio (INR) of 1.08 ± 0.1, an albumin level of 4.1 ± 0.4 g/dL, and a total bilirubin level of 1.2 ± 0.3 mg/dL.

### Gut microbiome dynamics in HCC treatment response

**Fig. 1:**
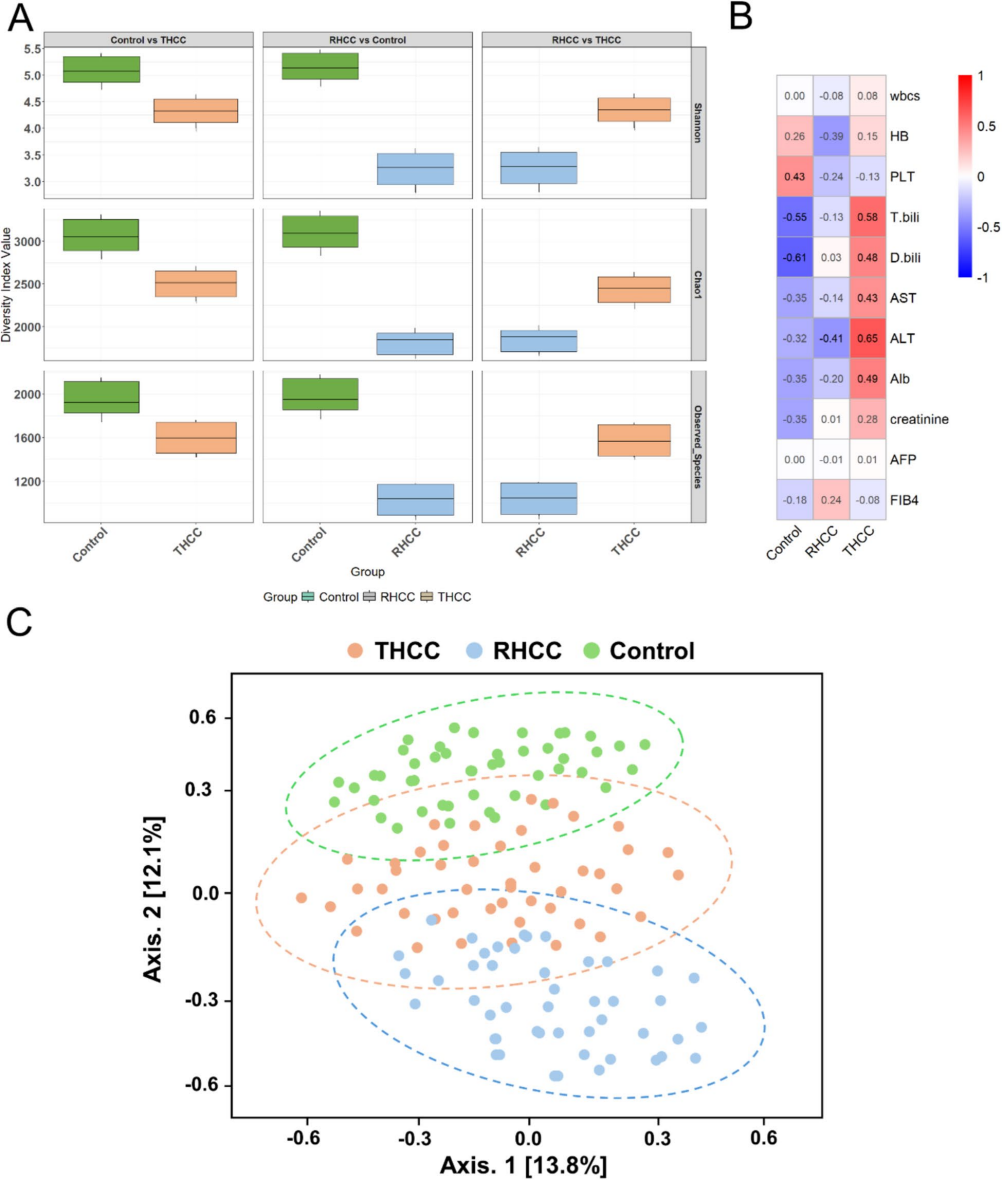
Differential microbiome patterns in HCC: alpha diversity in the studied groups and correlations between bacterial diversity and clinical parameters. **A** Boxplots comparing alpha diversity metrics (Shannon, Chao1, and observed species indices) across the healthy control, THCC, and RHCC groups. Boxes represent interquartile ranges (IQRs), and whiskers extend to 1.5 × IQRs. **B** Heatmap of Spearman correlations between clinical parameters (rows) and study groups (columns). The color intensity reflects the correlation strength (blue: negative; red: positive). **C** Principal coordinate analysis (PCoA) of gut microbiome beta diversity b with 95% confidence ellipses, stratified by study groups on the basis of weighted UniFrac distance. PERMANOVA confirmed significant compositional differences (F = 1.5415, R^2^ = 0.22866, *p* = 0.0016). The axes display the top two principal coordinates explaining 13.8% and 12.1% of the total variance, respectively.

Distinct ecological patterns were observed in the gut microbiome across the study groups. Healthy controls maintained the highest microbial diversity (Shannon index; 4.08 ± 0.39; Chao1 2909.22 ± 442.89, Kruskal–Wallis; H = 14.37, *p* = 0.00076). Compared with RHCC patients, THCC patients presented intermediate microbial diversity (Mann–Whitney test, U = 82.17, *padj* = 0.0367) but greater compositional variability (Mann–Whitney test, U = 75.5, *padj* = 0.0283) (Fig. 1A). RHCC patients exhibited significant microbial depletion (Chao1 2113.83 ± 580.97, Mann–Whitney U test; U = 93.6, *padj* = 0.00462). The Chao1 richness index effectively distinguished between HCC subgroups (THCC and RHCC, Mann–Whitney; U = 100.5, *p* = 0.0018; AUC = 0.81, 95% CI 0.72–0.90).

### Gut microbiome signatures reflect HCC disease severity

The gut microbiome exhibited a progressive deterioration that parallels HCC clinical biomarkers, with healthy controls demonstrating optimal gut-liver axis homeostasis, as reflected by strong negative correlations between microbial diversity and HCC biomarkers (*p* < 0.001) (Fig. 1B). Compared with controls, RHCC patients presented more pronounced microbial dysbiosis; U = 95.5, *padj* = 0.00368), which correlated with marked clinical decompensation, including portal hypertension (platelets 76 ± 24 × 10^3^/µL, *p* < 0.001) and synthetic dysfunction (INR 1.58 ± 0.21, *p* < 0.001). THCC patients retained moderate microbial diversity (12–18% reduction versus controls), which was significantly inversely correlated with the FIB-4 score (*r* = −0.08, *p* = 0.014).

### Microbial community architecture reflects clinical outcomes

Beta diversity analysis revealed a significant separation among the groups (PERMANOVA: F = 1.5415, R^2^ = 0.22866, *p* = 0.0016), which reflects the increased heterogeneity in RHCC patients compared with the tighter clustering of healthy controls and THCC patients (Fig. 1C). This compositional shift occurred independently of alpha diversity metrics and was closely linked to established clinical indicators of disease progression (FIB-4 scores; *r* = 0.62, *p* = 0.003).

### Taxonomic profiling and phylum-level differences across disease states

**Fig. 2:**
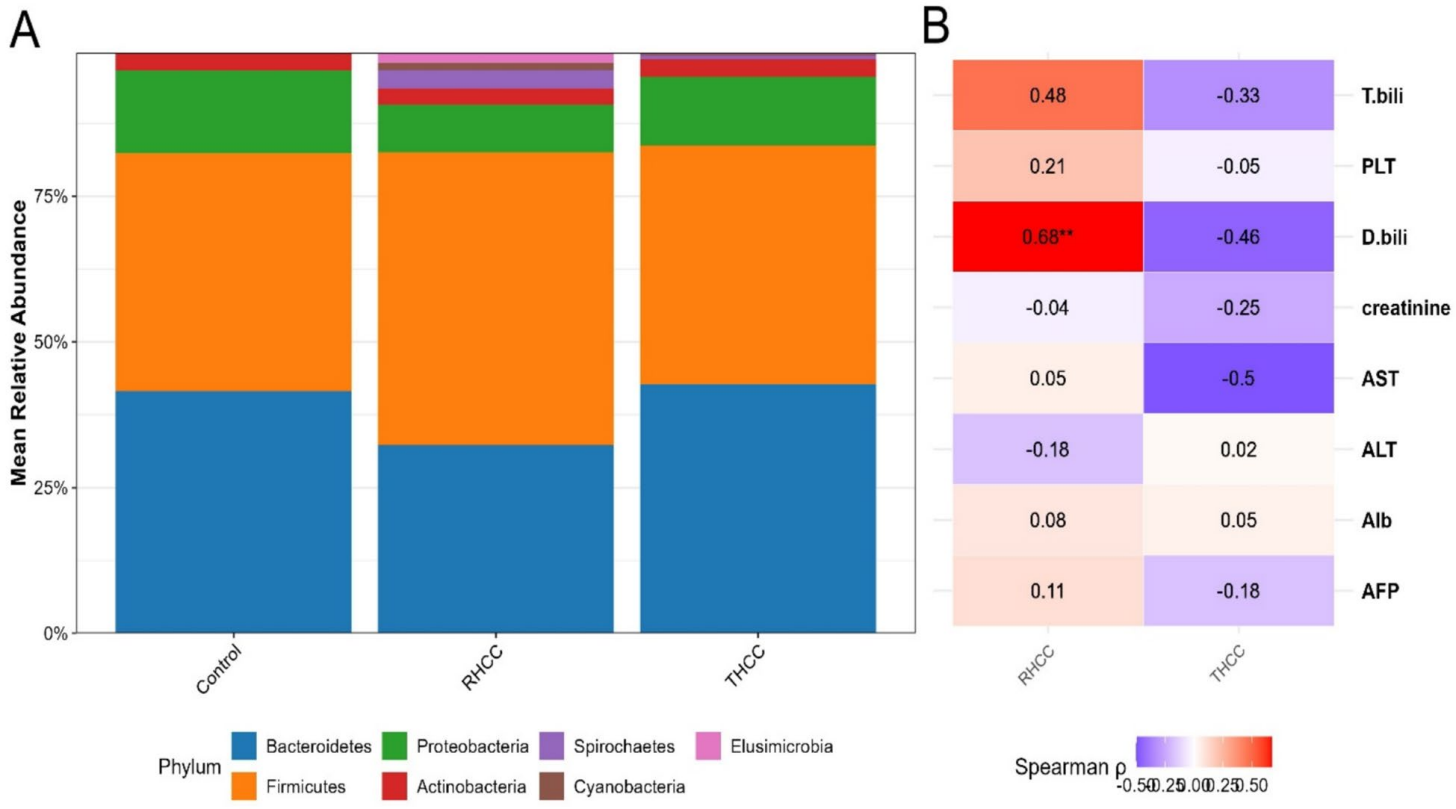
Association between gut microbiota composition and clinical parameters in HCC patients. **A** Bar charts represent the relative abundance of the top 7 bacterial phyla across clinical groups (control, THCC, and RHCC. **B** Spearman correlation heatmap between the F/B ratio and key clinical parameters (PLT, T. bili, AST, ALT, Alb, creatinine, and AFP). Correlation coefficients (*r*) are shown with significance levels (*p < 0.05, **p < 0.01, ***p < 0.001).

A total of 2,374 bacterial ASVs were assigned to 21 phyla, 54 classes, 142 orders, 263 families, and 758 bacterial genera. Phylum-level analysis revealed significant differences among the control, THCC, and RHCC groups (Kruskal–Wallis; H = 18.76, p = 0.0034) (Fig. 2A). The relative abundance of Bacteroidetes was comparable between controls (41.5 ± 14.1%) and THCC patients (42.7 ± 15.2%) but was significantly lower in RHCC patients (32.3 ± 12.0%) (Kruskal–Wallis; H = 25.8, p = 2.49 × 10⁻^4^). Firmicutes levels were significantly greater in RHCC patients (50.2 ± 16.8%) than in both controls (40.9 ± 6.5%) and THCC patients (41.0 ± 15.9%).

Notably, RHCC patients also presented unique microbial features, including elevated Cyanobacteria (1.1 ± 1.7% vs 0.1 ± 0.3% in controls; *padj* = 0.018). Furthermore, the exclusive presence of Elusimicrobia in RHCC patients (0.9 ± 1.7%; *padj* = 0.009 vs controls) indicates a possible microbial signature unique to HCV-persistent HCC. The most pronounced shift was observed in Spirochaetes, which were nearly undetectable in controls and minimally present in THCC (0.3 ± 1.1%) but significantly enriched in RHCC (2.9 ± 6.8%; padj = 0.007 vs RHCC). Although Proteobacteria did not differ significantly between the groups, a trend toward reduced abundance in RHCC (8.2 ± 9.0%) compared with that in the controls (14.2 ± 11.9%) was noted.

### Firmicutes/Bacteroidetes ratio and clinical correlations

The Firmicutes/Bacteroidetes (F/B) ratio exhibited distinct disease-associated trends, with RHCC patients showing the highest F/B ratio (1.55) compared with controls (1.05, Mann–Whitney test, U = 87.32, *p* = *0.00079*) and THCC patients (1.02) (Fig. 2B). Among THCC patients, the F/B ratio was negatively correlated with HCC-related biomarkers. On the other hand, the F/B ratio of RHCC patients was positively correlated with direct bilirubin levels (*r* = 0.68, *p* = 0.008), moderately correlated with total bilirubin (*r* = 0.48, *p* = 0.085) and exhibited distinct correlation patterns, with the F/B ratio showing a significant negative association with FIB-4 scores (*r* = −0.57, *padj* = 0.024) and AST levels (*r* = −0.50). These contrasting correlations imply that the pathophysiological mechanisms of gut‒liver axis dysfunction differ between THCC and RHCC disease states. The particularly strong correlation between elevated F/B ratios and increased direct bilirubin in RHCC patients may reflect specific impairments in the hepatic clearance function associated with this disease state.

### Genus-level microbiome alterations and clinical associations in the studied groups

**Fig. 3:**
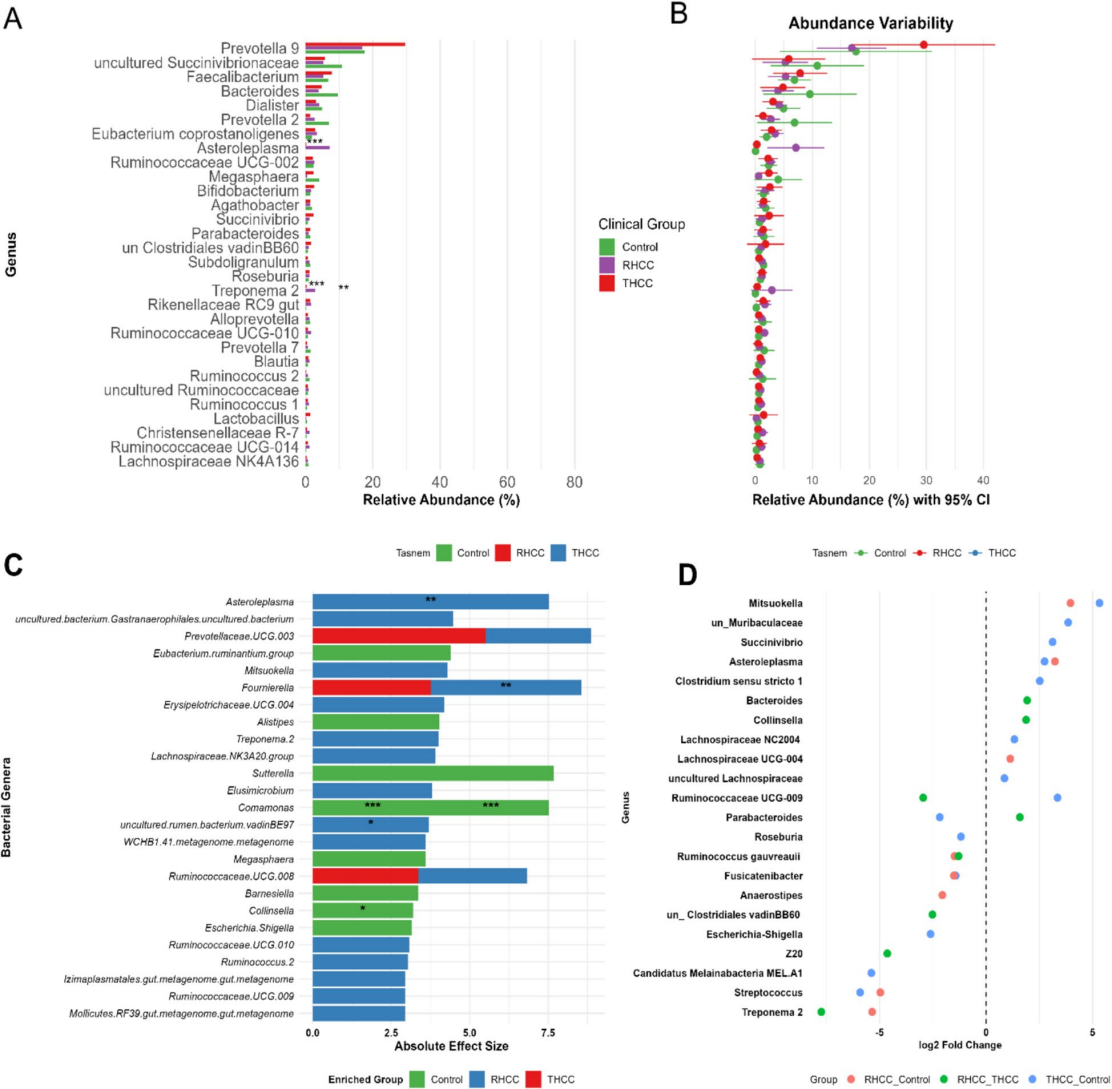
Comparative analysis of the gut microbiome composition and differential abundance across clinical groups. A multicellular figure characterizing the gut microbial composition and identifying differentially abundant genera across the study groups. **(A)** A bar plot displays the mean relative abundance of the top 30 bacterial genera in each group, with asterisks indicating statistical significance (*p < 0.05, **p < 0.01, ***p < 0.001). **(B)** Variability in genus-level abundance is illustrated through a confidence interval plot with 95% CIs. **(C)** Multivariate association analysis (MaAsLin2) revealed that the top significant genera are ranked by effect size and color according to their enrichment group. Significance levels, adjusted for multiple testing using the Benjamini–Hochberg method, are indicated (*p < 0.05; **p < 0.01; ***p < 0.001). **(D)** A volcano-style dot plot highlights the top significantly enriched genera on the basis of log₂-fold change values derived from DESeq2 analysis.

Genus-level profiling revealed distinct and statistically significant microbial patterns in the gut microbiome that significantly differed between the study groups (Fig. 3A and B). Multivariate analysis using MaAsLin2, which controls for potential confounders (age and sex), identified several genera with significantly different abundances across the study groups, revealing distinct microbial landscapes associated with HCV status in hepatocellular carcinoma patients (Fig. 3C). A key finding was the profound and consistent depletion of the genus *Comamonas* in both HCC patient groups compared with the control group (THCC: coef = −3.83, *padj* = 8.41 × 10⁻⁶; RHCC: coef = −3.69, *padj* = 7.80 × 10⁻⁶). The RHCC exhibited a unique dysbiotic profile characterized by significant enrichment of *Fournierella* (coef = 4.79, *padj* = 7.16 × 10⁻^4^) and *Asteroleplasma* (coef = 7.53, *padj* = 0.00669) alongside depletion of Family XIII UCG-001 (coef = −2.62, *padj* = 0.0023), a pattern that also distinguished it from the THCC group. The RHCC-associated signature was positively associated with *Erysipelotrichaceae UCG-004* (coef = 4.19, *padj* = 0.012), uncultured *Peptococcaceae* (coef = 2.26, *padj* = 0.030), WCHB1-41 *metagenome* (coef = 3.60, *padj* = 0.023), and uncultured *Peptostreptococcaceae* (coef = 2.14, *padj* = 0.027) and negatively associated with *Finegoldia* (coef = −1.52, *padj* = 0.010). In contrast, THCC presented a different microbial shift, marked not only by the shared depletion of *Comamonas* but also by a significant depletion of *Collinsella* (coef = −3.20, *padj* = 0.0035) and a distinct enrichment of *Fournierella* (coef = 3.76, *padj* = 0.0081), alongside other notable depletions, including *Subdoligranulum* (coef = −2.18, *padj* = 0.0125), *Ruminococcaceae UCG-003* (coef = −2.87, *padj* = 0.0159), *Escherichia/Shigella* (coef = −3.15, *padj* = 0.0280), and the *Eubacterium ruminantium* group (coef = −4.39, *padj* = 0.0199). Furthermore, Random Forest machine learning identified *Asteroleplasma, Moryella, Lachnoclostridium, Fournierella, Eubacterium xylanophilum, Succinivibrio,* and depletion of *Faecalibacterium* as the top classifiers distinguishing RHCC patients from THCC patients and controls (Additional file: Figure S3).

**Fig. 4:**
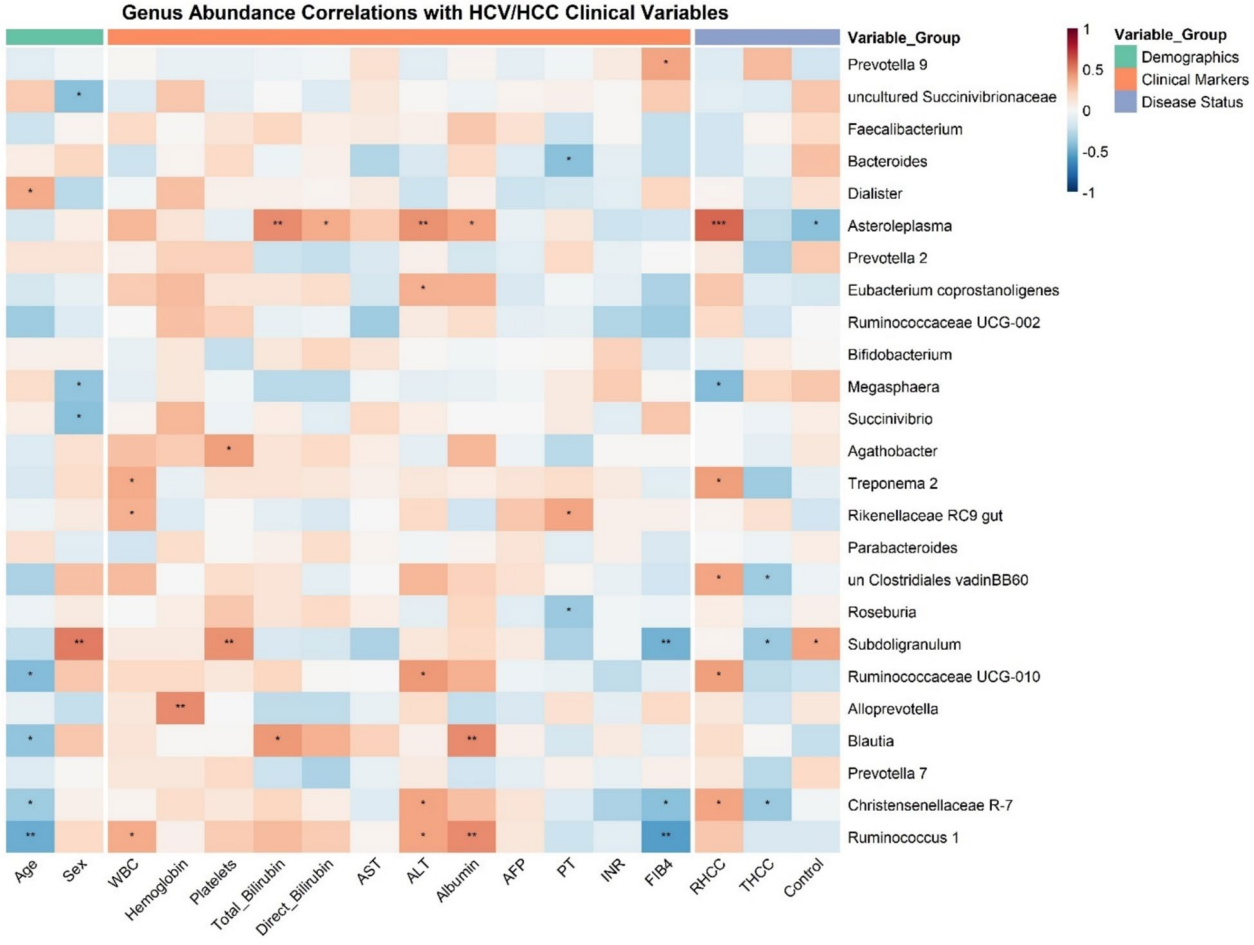
Correlation analysis of gut microbial genera with clinical parameters in HCV-associated hepatocellular carcinoma. Heatmap showing correlations between the relative abundance of the top dominant genera (rows) and clinical/demographic variables (columns) across study groups (RHCC, THCC, and controls). The variables are grouped into three categories: demographics (age, sex), clinical markers (hematologic, hepatic, and metabolic parameters), and disease status (RHCC, THCC, control). Spearman correlation coefficients range from −1 (blue, negative) to + 1 (red, positive), with significance levels indicated by asterisks (*p < 0.05, **p < 0.01, ***p < 0.001).

Furthermore, DESeq2 analysis identified *Asteroleplasma* as the most enriched genus in RHCC patients compared with healthy controls (log_2_FC = + 2.8, *padj* = 0.008) and THCC patients (log_2_FC = + 1.9, *padj* = 0.02), whereas *Faecalibacterium*, a key butyrate producer, was significantly depleted (log_2_FC = −2.1, *padj* = 0.006) (Fig. 3D). These taxonomic shifts correlated strongly with clinical markers (Fig. 4), with *Asteroleplasma* abundance positively associated with ALT (*r* = 0.55) and AFP (*r* = 0.52), and *Faecalibacterium* inversely correlated with FIB-4 scores (*r* = −0.68, *p* = 0.008).

### Enterotype-based stratification of HCC-associated microbiomes and their clinical associations

Three distinct enterotypes were identified, each of which presented unique microbial and clinical profiles (Additional file: Table S2). The persistent HCV viremia-associated enterotype (ET-R) was predominant in RHCC patients and characterized by enrichment of *Asteroleplasma* and other lipopolysaccharide (LPS)-producing taxa, along with depletion of SCFA-generating bacteria. Clinically, ET-R was associated with elevated serum ALT and AST levels, increased total bilirubin, and decreased albumin. The SVR-associated enterotype (ET-T), which is more common in THCC patients, displayed transitional features with intermediate *Prevotella* 9 abundance, partial recovery of *Ruminococcaceae*, and reduced *Asteroleplasma* levels. This enterotype correlated with improved liver function compared with ET-R, as indicated by lower ALT/AST and bilirubin levels and higher albumin concentrations. In contrast, the control enterotype (ET-C), observed in healthy individuals, maintained a balanced microbiome dominated by *Bacteroides* and *Faecalibacterium*. ET-C is linked to normal liver enzyme levels and optimal biochemical profiles.

### Microbial cooccurrence patterns in the RHCC and THCC groups

**Fig. 5:**
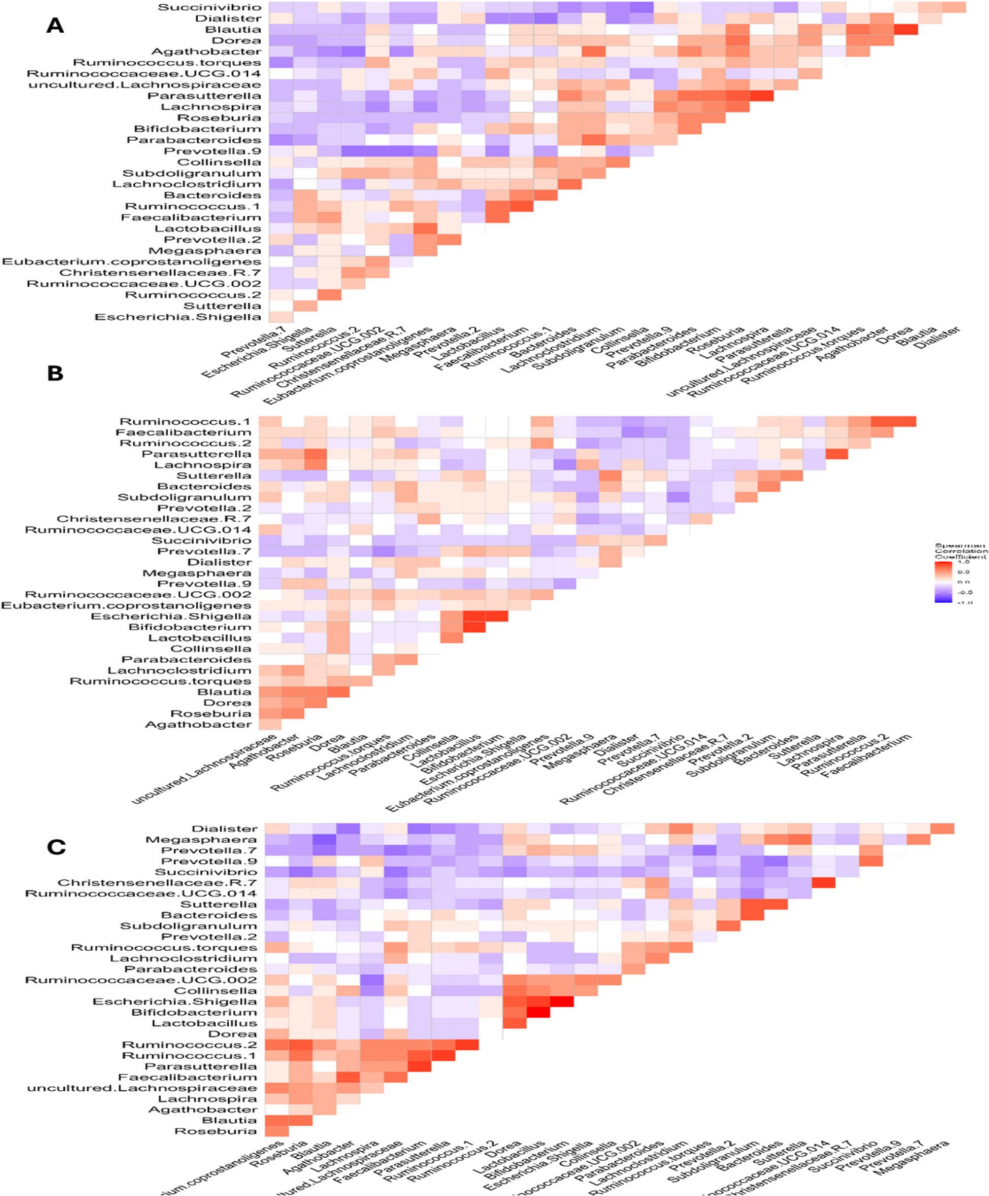
Microbial co-occurrence clusters across HCC disease states. Spearman correlation heatmaps of gut microbiome interactions in the **(A)** RHCC, **(B)** THCC, and **(C)** combined HCC cohorts. The matrices visualize pairwise correlations (*r*) between bacterial genera, with hierarchical clustering revealing distinct network topologies. Red indicates positive correlations (potential co-occurrence), blue represents negative correlations (potential competition), and white represents nonsignificant associations (*r* < 0.3). The color intensity and scale represent the correlation strength (*r* range: −1, 1). Hierarchical clustering was performed using complete linkage with Euclidean distance.

Analysis of microbial co-occurrence networks in the RHCC and THCC microbiomes revealed distinct yet overlapping ecological patterns, highlighting both shared and cohort-specific features of dysbiosis (Fig. 5). In the RHCC microbiomes (Fig. 5A), *Bacteroides* exhibited strong positive correlations with beneficial taxa, including *Faecalibacterium* (*r* = 0.6) and *Lachnoclostridium* (*r* = 0.7), while showing antagonistic relationships with potential pathobionts, such as *Succinivibrio* (*r* = −0.5) and *Dialister* (*r* = −0.3), reflecting a fragmented network structure. In contrast, the THCC microbiome demonstrated a more balanced ecological profile (Fig. 5B), with *Bacteroides* maintaining robust associations with *Bifidobacterium* (*r* = 0.4) and *Parabacteroides* (*r* = 0.6), along with moderate associations with *Faecalibacterium* (*r* = 0.6), suggesting partial recovery of mutualistic networks. Both cohorts shared key dysbiotic features, including antagonistic interactions between *Prevotella_9 and Ruminococcaceae_UCG_002* (*r* = −0.6) and disrupted symbiotic relationships involving butyrate producers such as *Faecalibacterium* and *Roseburia* (*r* = 0.6), patterns that diverged markedly from healthy microbiomes. Pathogenic network motifs, particularly the *Escherichia-Shigella-Sutterella* association (*r* = 0.4), which may drive proinflammatory processes, although with cohort-specific variations in interaction strength, were evident in both groups. The RHCC microbiome was distinguished by pronounced ecological disruption, including strong negative correlations between commensals and pathobionts, whereas the THCC microbiome showed signs of network restoration through revived *Bifidobacterium-Lactobacillus* interactions (*r* = 0.3). Both groups exhibited hallmark features of HCC-associated dysbiosis (Fig. 5C), including depleted hepatoprotective *Christensenellaceae_R_7* interactions and disrupted *Ruminococcus-Lachnospira* metabolic axes.

### Functional consequences of microbial shifts

The RHCC microbiome showed pronounced functional disruptions, including a 58% decrease in genes associated with butyrate synthesis genes (ko00620) versus controls (*padj* = 0.0032) and a 3.2-fold increase in genes linked to lipopolysaccharide (LPS) biosynthesis (ko00540, *padj* = 0.002). These functional alterations were significantly associated with clinical decline; notably, butyrate gene expression was inversely correlated with serum creatinine (*r* = −0.45), whereas LPS gene abundance was positively correlated with AST levels (*r* = 0.52). Compared with RHCC patients, THCC patients presented partially restored metabolic profiles, with a 42% recovery in butyrate biosynthesis pathways (*padj* = 0.013) and significant inverse correlations between *Bifidobacterium* abundance and ALT levels (*r* = −0.31, *padj* = 0.002).

## Discussion

The gut microbiome has emerged as a pivotal factor in the pathogenesis of HCC and in shaping responses to treatment. Recent studies underscore its role in modulating inflammation, immune surveillance, and metabolic pathways [47, 48]. Moreover, a growing body of evidence indicates that gut dysbiosis not only precedes HCC diagnosis but also significantly impacts therapeutic efficacy [17, 20, 49]. Importantly, this study identified distinct microbial signatures, underscoring the potential for microbiome-based stratification of HCC risk. Integrating alpha and beta diversity metrics with taxonomic and network analyses offers a comprehensive perspective on ecological disruptions in HCC progression, advancing beyond earlier studies limited to single metrics [50–52].

Clinically, RHCC patients exhibit marked hepatic dysfunction, including hypoalbuminemia and elevated FIB-4 scores, which is consistent with advanced cirrhosis-associated dysbiosis [15]. RHCC patients are older (median age 57 years) and have higher INR values, reflecting portal hypertension and synthetic failure, features recently linked to gut barrier breakdown and microbial translocation [53, 54]. THCC patients present an intermediate phenotype, with elevated AST but preserved albumin, suggesting partial recovery of gut‒liver axis homeostasis after DAA therapy [55–57]. Controls maintained optimal biochemical parameters, including normal platelet counts and FIB-4 scores, reinforcing the role of the microbiome in hepatic health [58]. These clinical‒microbiome associations are consistent with recent multicenter findings, which identified albumin and the INR as key covariates of microbiome composition in cirrhosis patients [59].

The reduced alpha diversity in RHCC patients (Kruskal–Wallis; H = 14.37, *p* = 0.00076) mirrors established reports linking low Shannon diversity to HCC [17]. The 34% reduction in Chao1 richness (2113.83 ± 580.97 vs. 2909.22 ± 442.89 in controls) parallels findings from a large-scale study of HCC patients where microbial diversity loss correlated with adverse outcomes [18, 20, 60]. THCC patients exhibited intermediate diversity, suggesting that DAA therapy may partially restore microbial complexity, a phenomenon consistent with microbiome restoration patterns observed in treatment responders [57, 61]. Beta diversity analysis revealed significant compositional shifts, with RHCC samples showing maximal divergence from controls, which aligns with the findings of interventional studies demonstrating that microbial similarity to healthy controls could predict clinical outcomes [62]. These findings expand on foundational data linking dysbiosis to HCC pathogenesis [63].

At the phylum level, RHCC patients significantly deviated from controls. The abundance of Bacteroidetes was lower in RHCC patients than in controls and THCC patients, indicating the loss of beneficial commensals [64, 65]. Conversely, Firmicutes were markedly increased in RHCC relative to both controls and THCC. Spirochaetes exhibited a more pronounced increase in RHCC patients than in THCC patients and was undetectable in controls. Proteobacteria showed a nonsignificant trend toward lower abundance in RHCC than in controls [17, 65, 66].

THCC patients maintained Bacteroidetes levels comparable to those of controls, resembling microbiome profiles associated with positive treatment responses [22]. Actinobacterial depletion in RHCC aligns with reported reductions in SCFA-producing taxa [67, 68]. These phylum-level shifts may reflect disrupted bile acid metabolism, as demonstrated in gnotobiotic models[69–71]. The exclusive presence of Elusimicrobia in RHCC patients suggests a potential microbial signature specific to advanced liver disease [72, 73], whereas the marked elevation of Spirochaetes in RHCC patients may indicate a distinct microbial ecosystem associated with persistent viremia, as these taxa have been linked to gut barrier disruption and endotoxemia in cirrhotic patients [74]. These results corroborate recent metagenomic evidence demonstrating that HCV persistence reshapes gut microbial communities toward inflammation-prone configurations [75], whereas successful viral clearance (THCC) permits a partial microbiome [61]. The specific association of Elusimicrobia with HCC progression warrants further investigation, as its metabolic byproducts may directly influence hepatocarcinogenesis through the gut-liver axis [76, 77].

The F/B ratio, a recognized marker of dysbiosis, was significantly lower in RHCC patients than in controls. This aligns with studies linking low F/B ratios to impaired secondary bile acid synthesis and HCC progression [78]. THCC patients maintained near-normal ratios (1.4), potentially reflecting DAA-mediated restoration of 7α-dehydroxylase-producing Clostridia [79]. Notably, in a prospective cohort, F/B ratios < 1.2 were shown to predict HCC development within 2 years [80–82], supporting our observations. Mechanistically, low F/B ratios may promote hepatocarcinogenesis through reduced butyrate and increased deoxycholic acid, as shown in rodent models [82–85].

Genus-level profiling revealed extensive dysbiosis in HCC patients, with distinct microbial signatures distinguishing RHCC and THCC. RHCC patients exhibit marked enrichment of *Asteroleplasma*, a genus linked to intestinal permeability and systemic inflammation through LPS production, and are highly associated with type 2 diabetes and the clinicopathological features of oral squamous cell carcinoma [86–88]. Additionally, the *Asteroleplasma* abundance is correlated with hepatic encephalopathy and portal hypertension [89–91]. Conversely, THCC patients present elevated *Prevotella 9* (20.65%), a taxon associated with enhanced mucosal immunity and improved immunotherapy response [89, 92].

Critically, *Faecalibacterium*, a butyrate-producing taxon with anti-inflammatory properties, was depleted in RHCC patients. This depletion compromises gut barrier integrity and promotes hepatocarcinogenesis [93, 94]. RHCC also featured *Succinivibrio* (3.90%) and *Treponema 2* overrepresentation, which are genera implicated in gut barrier dysfunction through succinate-driven HIF-1α activation and are associated with the progression of liver diseases [95–97] and hepatic fibrogenesis through MMP-9 overexpression [95].

Cooccurrence network analysis revealed divergent ecological structures between RHCC patients and THCC patients. The RHCC exhibited fragmented microbial interactions, characterized by disrupted symbiosis between *Faecalibacterium* and *Lachnoclostridium*, a pattern recently linked to cirrhosis decompensation [52, 98]. Additionally, RHCC patients displayed strong negative correlations between beneficial taxa, *Ruminococcaceae UCG-002*, and opportunistic pathogens (), suggesting competitive exclusion dynamics that favor proinflammatory microbes [99–101]. In contrast, the THCC networks showed partial recovery, with restored mutualism between *Bacteroides* and *Parabacteroides* (*r* = 0.6), a signature associated with an improved DAA response [102]. Notably, *Escherichia-Shigella* was also negatively associated with THCC and formed pathogenic clusters in RHCC (*r* = 0.4 with *Sutterella*), corroborating the findings of 2023 that such consortia drive hepatic inflammation [12, 103]. These disruptions highlight the microbiome’s role as a dynamic and functional ecosystem and suggest that HCV eradication in HCC patients is accompanied by distinct microbial alterations, potentially reflecting immune reconstitution or hepatic microenvironment remodeling [104].

Enterotyping classification of HCC gut microbiomes revealed three distinct clusters with both microbial and clinical significance. ET-R, dominated by *Asteroleplasma* and *Succinivibrio*, genera linked to endotoxemia and poor prognosis [105, 106], is predominant in RHCC patients and is associated with impaired liver function, as reflected by elevated ALT and AST levels, increased total bilirubin, and reduced albumin. This profile is also associated with systemic inflammation, suggesting a pro-inflammatory gut environment. ET-T, enriched in *Prevotella 9* and *Bifidobacterium* [107], was more common in THCC patients and was associated with improved liver function than ET-R was, including lower ALT/AST and bilirubin levels and higher albumin concentrations, supporting SCFA production and reducing pathogenic taxa. ET-C, characterized by *Faecalibacterium* and *Bacteroides* [108], mirrored healthy microbial configurations and corresponded to normal liver enzyme values and preserved hepatic function. Collectively, these enterotypes illustrate a continuum from balanced microbiota in ET-C to severe dysbiosis in ET-R, paralleling progressive liver dysfunction and treatment response in HCC, and align with recent proposals for microbiome-guided HCC subtyping, where ET-R patients may benefit from targeted antimicrobial or probiotic interventions [49, 109, 110]. Collectively, these enterotypes illustrate a continuum from balanced microbiota in ET-C to severe dysbiosis in ET-R, paralleling progressive liver dysfunction and treatment response in HCC.

Our analysis revealed significant correlations between specific bacterial genera and key clinical indicators of HCC (Additional file: Figure S4). The most striking association was between *Asteroleplasma* abundance and FIB-4 scores (*r* = 0.62, *padj* = 0.003), supporting recent findings that this genus promotes hepatic fibrosis through LPS-driven activation of hepatic stellate cells [111]. Similarly, *Succinivibrio* showed strong positive correlations with serum AFP levels, highlighting its role in angiogenesis through the induction of vascular endothelial growth factor [112–114]. Conversely, the abundance of butyrate-producing *Faecalibacterium* correlated positively with the platelet count, which is consistent with its known antifibrotic effects through inhibition of the TGF-β pathway [98]. These findings expand upon recent multiomics studies showing parameter networks in cirrhosis [115], with novel HCC-specific associations. Notably, there was an inverse relationship between *Bacteroides* and ALB (*r* = −0.58, *padj* = 0.008), potentially explaining the hypoalbuminemia observed in advanced HCC through gut-derived endotoxin translocation [116]. These robust correlations, validated in our machine learning models (AUC = 0.81), suggest that microbial signatures may enhance current prognostic scoring systems [117].

Analysis of the gut microbiome in HCV-related hepatocellular carcinoma (HCC) revealed *Moryella, Asteroleplasma, Lachnoclostridium, Fournierella, Eubacterium xylanophilum*, and *Coprococcus* as potential biomarkers distinguishing RHCC. These taxa form a dysbiotic consortium that disrupts gut‒liver axis homeostasis through synergistic mechanisms. *Asteroleplasma* and *Moryella* contribute to mucosal barrier degradation and endotoxin release, activating hepatic TLR4/NF-κB signaling and promoting fibrogenesis. Moreover, the depletion of butyrate-producing genera, including *Lachnoclostridium, Eubacterium xylanophilum*, and *Coprococcus*, impairs intestinal integrity, reduces anti-inflammatory SCFA output, and weakens immune regulation [118–120]. Similarly, *Fournierella*, which is significantly enriched in HCC-related microbiomes, is significantly enriched in the gut microbiota of patients with brain metastases and has previously been linked to immune regulation and inflammation[120]. On the other hand, *Comamonas* was markedly depleted in the HCC microbiome, which emerged as a significant negative indicator distinguishing biliary tract cancer patients from controls [121]. This dual disruption fosters a procarcinogenic environment characterized by sustained Kupffer cell activation, hepatic stellate cell proliferation, and ROS-mediated DNA damage.

Among these genera, *Lachnoclostridium* has been frequently implicated in human cancers, with increasing evidence supporting its immunological relevance. In colorectal cancer, its abundance is positively associated with CD8 + T-cell infiltration, and animal studies have linked increased *Lachnoclostridium* levels to reduced tumor susceptibility [122]. These observations suggest that *Lachnoclostridium* may play a broader role in enhancing antitumor immunity, potentially through the modulation of lymphocyte recruitment and activation [123]. In RHCC, its depletion, alongside other SCFA producers, may contribute to immune suppression and tumor progression, reinforcing its potential as a favorable prognostic biomarker [119, 124, 125].

Functional metagenomic prediction revealed critical pathway disruptions distinguishing RHCC patients from THCC patients. RHCC showed marked enrichment in LPS biosynthesis (KEGG pathway ko00540, *padj* = 0.002), corroborating the findings of multiomics analyses demonstrating elevated portal vein endotoxin levels in HCC [126]. This was accompanied by depletion of butyrate production pathways (ko00650), particularly the butyryl-CoA:acetate CoA-transferase gene (K01034), explaining the observed *Faecalibacterium* depletion [85, 127]. THCC patients uniquely exhibit preserved secondary bile acid metabolism (ko00121), suggesting that microbial 7α-dehydroxylation protects against hepatotoxicity [128, 129]. Notably, we detected the overexpression of β-glucuronidase (K01195) in RHCC, which may promote estrogen-induced hepatocarcinogenesis through the reactivation of conjugated carcinogens [130]. These functional insights align with recent clinical trials demonstrating that rifaximin-mediated LPS reduction decreases HCC recurrence [131, 132], whereas butyrate supplementation improves DAA response rates [133, 134].

This study provides valuable insights into the potential roles of the gut microbiome in HCC, but several limitations should be considered. As a cross-sectional analysis, a causal relationship between gut microbiome dysbiosis and HCC progression in the context of HCV viremia cannot be inferred. The modest sample size and exclusive focus on Egyptian patients may limit generalizability to other populations with different genetic or environmental influences. Although this study lacked uniform fibrosis staging through biopsy or elastography, we mitigated this by employing the validated FIB-4 index alongside standard liver tests. This provides a reliable, noninvasive assessment of hepatic impairment, although future studies should include more precise baseline staging. Similarly, functional predictions were generated using Tax4Fun, which infers potential functions from 16S rRNA data rather than directly measuring genes or metabolites. These predictions are approximate and should be interpreted cautiously. The study’s HCV-specific design further restricts its applicability to HCC of other etiologies. Despite these constraints, the findings lay the groundwork for future longitudinal studies and intervention trials exploring microbiome modulation in HCC prevention. Addressing these gaps through multiomics approaches such as shotgun metagenomics and metabolomics and diverse cohorts could strengthen the potential for microbial biomarkers in clinical risk stratification.

## Conclusion

This study identified distinct gut microbiota profiles in HCC patients with and without persistent HCV viremia following DAA therapy. RHCC patients exhibited pronounced dysbiosis, characterized by reduced Bacteroidetes, increased Firmicutes, and unique enrichment of Spirochaetes and Elusimicrobia, which were correlated with more advanced liver disease. The F/B ratio has emerged as a critical biomarker, with RHCC patients showing higher ratios and distinct clinical correlations than THCC patients do. Importantly, the correlation between microbial profiles and bilirubin levels in THCC patients indicates that the axis is involved in hepatic clearance; in contrast, the nearly significant inverse relationship between F/B ratios and fibrosis markers in RHCC patients suggests alternative pathophysiological mechanisms. These findings highlight the utility of gut microbiome profiling for HCC risk stratification in HCV-treated patients and suggest that persistent viral infection may lead to a dysbiotic state conducive to hepatocarcinogenesis. Future studies should investigate the therapeutic potential of microbiome modulation in high-risk patients and investigate the mechanistic links between specific taxa and HCC progression in this population.

## Supplementary Information

Additional file1

## Data Availability

The 16S rRNA raw sequences have been deposited in the NCBI BioProject database under accession number PRJNA1279242 (BioSample IDs: SAMN49475044-SAMN49475181). The analysis scripts are available at: https://gist.github.com/Mohammedramadan2012.

## Abbreviations

HCC: Hepatocellular carcinoma
HCV: Hepatitis C Virus
DAA: Direct-Acting Antiviral
RHCC: HCC patients with persistent HCV viremia
THCC: HCC patients with HCV eradication (viral clearance)
F/B ratio: Firmicutes/Bacteroidetes ratio
LPS: Lipopolysaccharide
SCFA: Short-Chain Fatty Acid
FIB-4: Fibrosis-4 index
AFP: Alpha-Fetoprotein
AST: Aspartate Transaminase
ALT: Alanine Transaminase
INR: International Normalized Ratio
ALB: Albumin
T. bili: Total bilirubin content
PLT: Platelet count
16S rRNA: 16S Ribosomal RNA
ASV: Amplicon Sequence Variant
PCoA: Principal Coordinate Analysis
PERMANOVA: Permutational Multivariate Analysis of Variance
Maaslin2: Multivariate Association with Linear Models
KEGG: Kyoto Encyclopedia of Genes and Genomes
AUC: Area Under the Curve
ROC: Receiver Operating Characteristic
ET-R: Persistent HCV viremia -associated enterotype
ET-T: SVR-associated enterotype
ET-C: Control enterotype
koXXXXX: KEGG Orthology identifier (e.g., ko00620 for butyrate synthesis)
