# Supplementary material for: Gut microbiome dysbiosis in hepatocellular carcinoma patients with persistent HCV viremia versus viral clearance: a cross-sectional study
Source: Gut Pathog. 2025 Nov 14;17:88. doi: 10.1186/s13099-025-00761-w (PMC12619186; doi:10.1186/s13099-025-00761-w)
Supplement: Supplementary file 1 — Additional file1 [file 13099_2025_761_MOESM1_ESM.pdf]

Gut Microbiome Dysbiosis in Hepatocellular Carcinoma Patients with Persistent HCV Viremia versus Viral Clearance: A Cross-Sectional Study

Hany R. Hashem[1 †], Tasnem Yehia[2 †], Marwa Azab[2], Ali Abdellah[2], Ibrahim A. Amin[3], Mohammed Salah[4], Mohammed Ramadan[3]*

Table S1. Statistical Summary of Classification Error Rates

Group	Mean Error	Min Error	Max Error	Std. Dev.
**Overall**	0.208	0.103	0.300	0.059
**Control**	0.110	0.053	0.196	0.041
**RHCC**	0.254	0.165	0.349	0.051
**THCC**	0.309	0.205	0.398	0.055

The control group had the lowest average error and least variability, indicating high classification accuracy. The THCC group had the highest average error, suggesting that classification was the most challenging. The overall error decreased and stabilized as the number of trees increased, indicating model convergence. Annotations highlight key points such as the lowest errors and convergence behavior.

Table S2. Microbiome Stratification by Enterotyping in HCC

**Enterotype**	**Key Genera (Mean Relative Abundance %)**	**Group Association**	**Significant Features**
ET-R (Relapse-associated)	1. Asteroleplasma<br>2. uncultured Succinivibrionaceae<br>3. *Succinivibrio*<br>4. Treponema 2<br>5. uncultured bacterium Clostridiales vadinBB60	RHCC	• Highest Asteroleplasma (KW *p*=0.009)<br>• Elevated LPS-producers<br>• Depleted SCFA-generators
ET-T (Treatment-associated)	1. Prevotella 9<br>2. *Bifidobacterium*<br>3. *Ruminococcaceae UCG-002*<br>4. Parabacteroides<br>5. Ruminococcaceae UCG-014	THCC	• Intermediate Prevotella 9 levels<br>• Partial Ruminococcaceae recovery<br>• Reduced Asteroleplasma
ET-C (Control-associated)	1. Faecalibacterium<br>2. Bacteroides<br>3. Megasphaera<br>4. Prevotella 2<br>5. Subdoligranulum	Control	• Highest Bacteroides (KW *p*=0.041)<br>• Balanced SCFA-producers<br>• Lowest pathogenic genera

Figure S1. Bacterial diversity analysis: Alpha diversity

Rarefaction curves demonstrating observed amplicon sequence variants (ASVs) across sequencing depths for (A) healthy versus diseased samples and (B) different disease statuses (Control/THCC/RHCC). Curves represent individual samples. The dashed vertical line indicates the rarefaction depth (27,119 reads) used for downstream analyses.

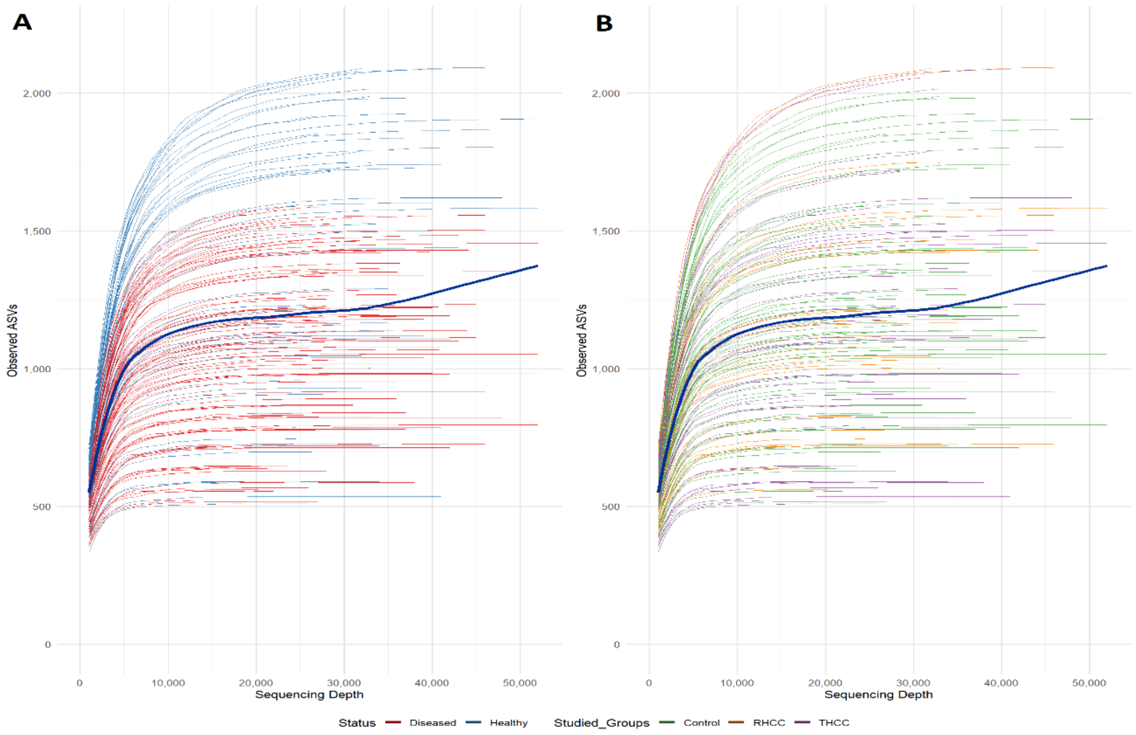

Figure S1. Variable-Length Rarefaction Curves Showing Microbial Diversity by Health and Diseased status

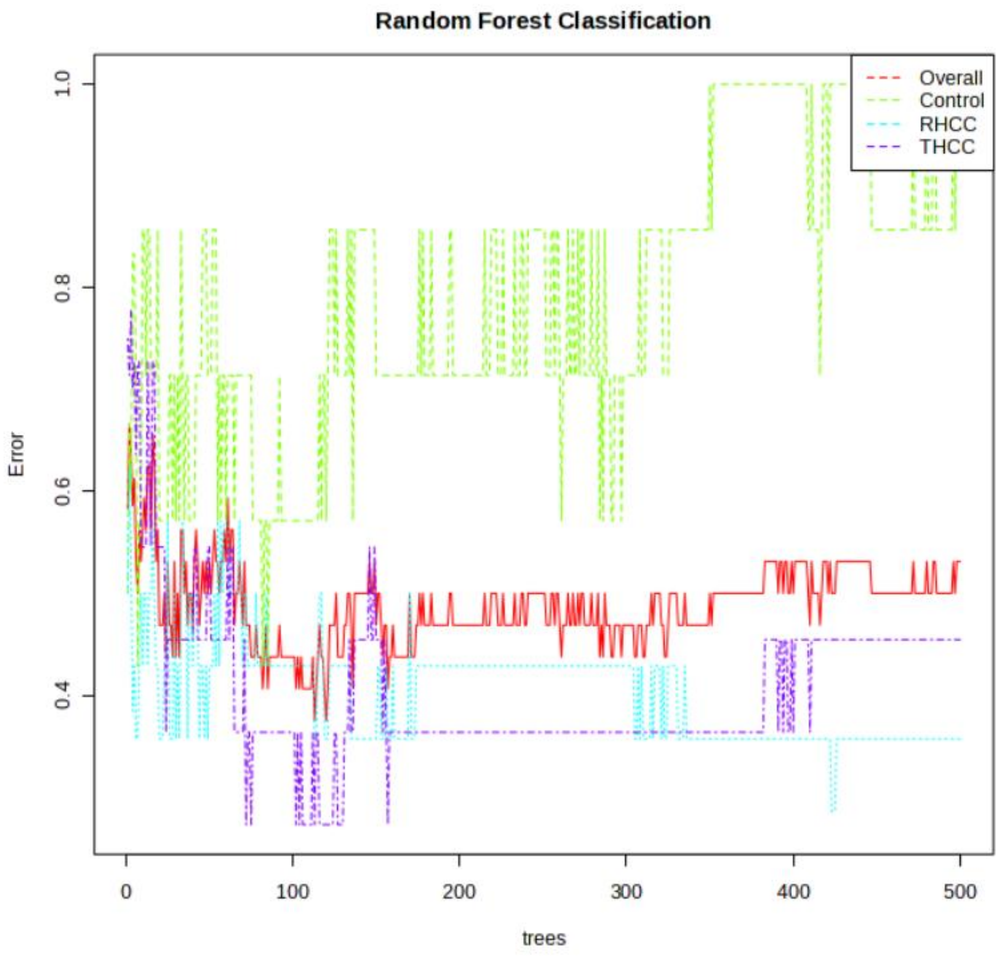

Figure S2. RF classification illustrates how the classification error rate changes as the number of trees in the RF model increases (from 0-500). As the number of trees increases, the error rates for all groups generally decrease, indicating improved model performance with more trees. The overall error stabilizes at a lower level, suggesting good general classification performance. The Control group appears to have the lowest error, indicating that it is the easiest to classify. The RHCC and THCC groups have higher error rates, possibly because of overlapping features or more complex patterns.

Figure S3. ROC Curves for Top Discriminative Bacterial Genera Across RHCC, THCC, and Control Groups.

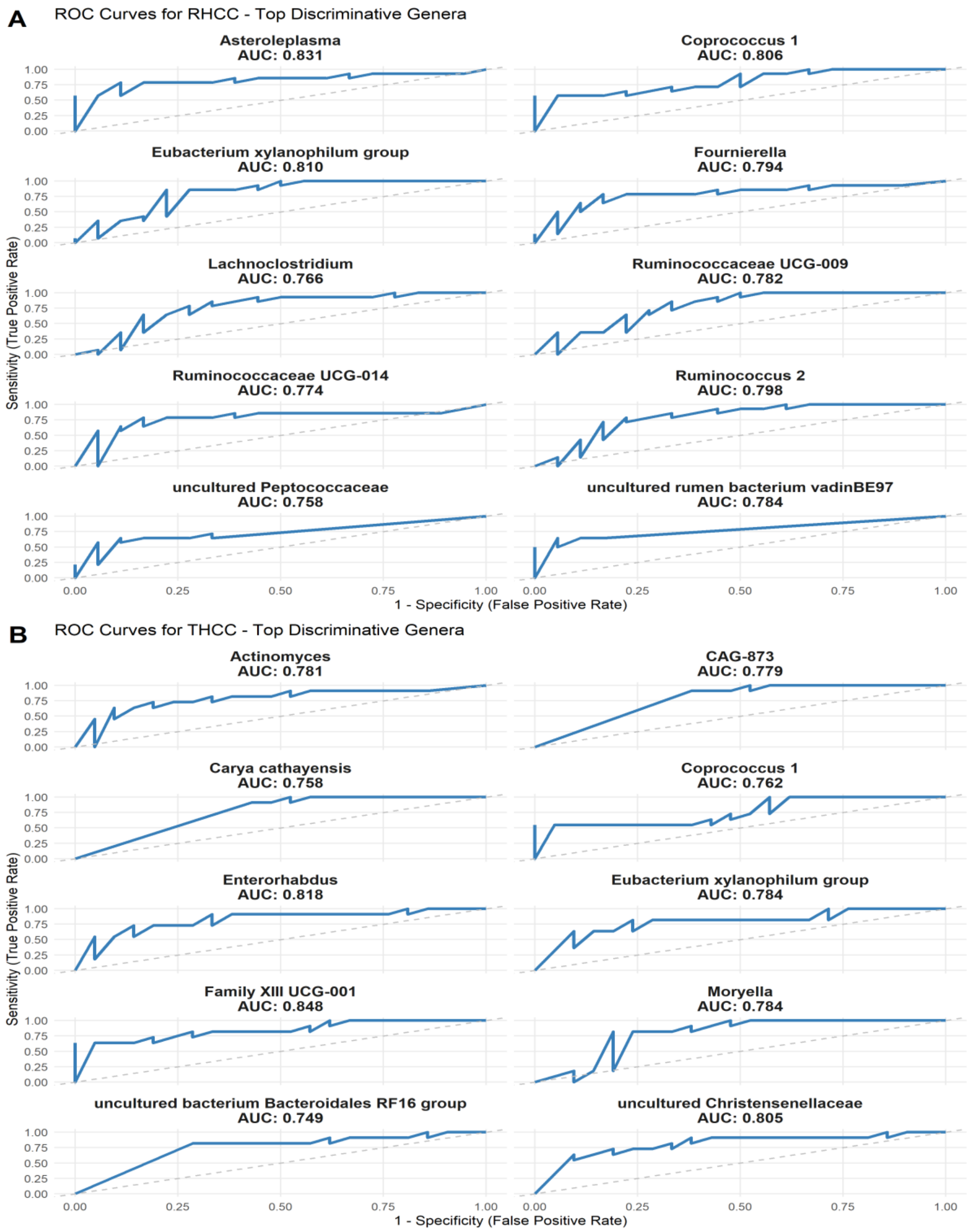

Figure S3. ROC Curves for Top Discriminative Bacterial Genera Across RHCC and THCC against Control Group

This figure displays Receiver Operating Characteristic (ROC) curves for the most discriminative bacterial genera identified in three clinical groups: (A) Top discriminative bacterial genera across RHCC against control group, (B) Top discriminative bacterial genera across THCC against control group. Each subplot illustrates the diagnostic performance of a specific genus, with sensitivity plotted against 1-specificity. The Area Under the Curve (AUC) values are provided to quantify the classification accuracy of each genus in distinguishing between groups. Genera such as *Asteroeplasma*, *Enterorhabdus*, and *Coprococcus_1* demonstrated high discriminative power across different comparisons.

Figure S4. Proposed Gut, Liver Axis Mechanisms in HCC After HCV Eradication: Role of Enterotypes.

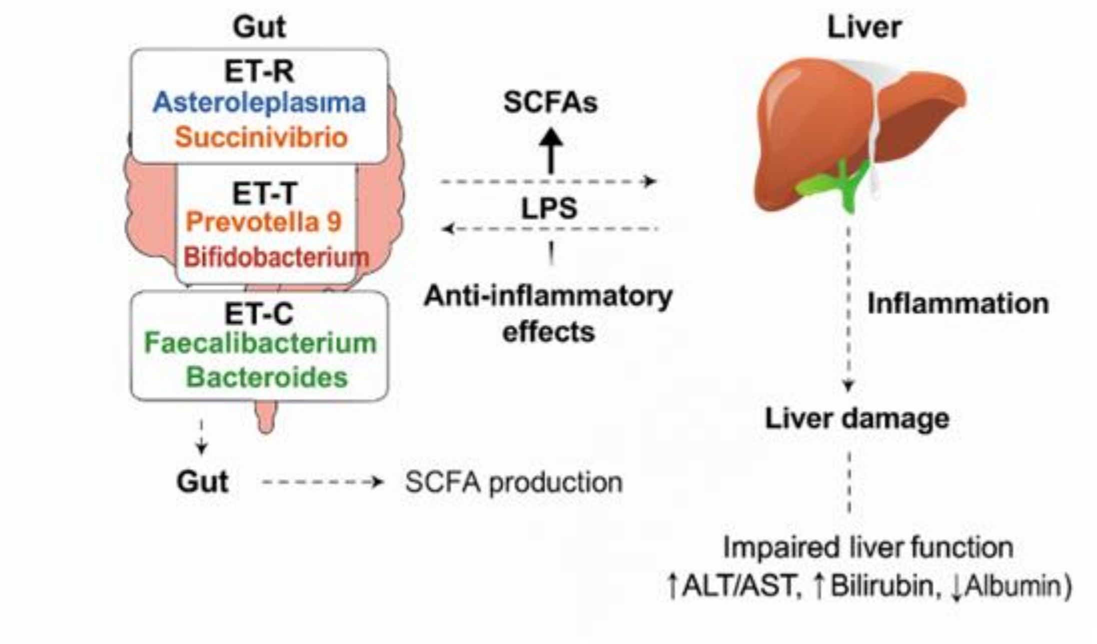

Figure S4. Proposed Gut, Liver Axis Mechanisms in HCC After HCV Eradication: Role of Enterotypes.

Schematic diagram illustrates the gut, liver axis in hepatocellular carcinoma (HCC) patients following HCV eradication, highlighting three enterotypes (ET-R, ET-T, ET-C) and their microbial and clinical potential characteristics. Enterotypes reflect a continuum from balanced microbiota (ET-C) to severe dysbiosis (ET-R), paralleling progressive liver dysfunction and treatment response. The liver and intestine icons used in this Figure were obtained from Pixabay (https://pixabay.com) and are free for use without attribution under the Pixabay license(https://pixabay.com/service/license/).
